# Physical Spacing and Social Interaction Before the Global Pandemic

**DOI:** 10.1007/s40980-021-00100-y

**Published:** 2021-10-13

**Authors:** Mathew Creighton, Daniel Capistrano, Agnieszka Sorokowska, Piotr Sorokowski

**Affiliations:** 1 School of Sociology, University College, Dublin, Ireland; 2 School of Education, University College Dublin, Dublin, Ireland; 3 Institute of Psychology, University of Wroclaw, Wroclaw, Poland

**Keywords:** Interpersonal distance, Social distance, Social interaction, SARS-CoV-2, COVID-19, Coronavirus

## Abstract

Subsequent to the arrival of SARS-CoV-2 and emergence of COVID-19, policy to limit the further spread has focused on increasing distance between individuals when interacting, often termed social distancing although physical distancing is more accurate (Das Gupta and Wong in Canadian J Public Health 111:488–489, 2020; Gale in Is ‘social distancing’ the wrong term? Expert prefers ‘physical distancing,’ and the WHO agrees. The Washington Post, 2020; Sørensen et al. in Glob Health Promot, 28:5–14, 2021), and limiting the frequency of interaction by limiting/prohibiting non-essential and large-scale social gatherings. This research note focuses on social spacing, defined by distance and interaction, to offer a cross-cultural insight into social distancing and social interactions in the pre-pandemic period. Combining unique data on frequency of contact, religious service attendance and preferred interpersonal spacing in 20 countries, this research note considers variation in the extent to which physical distance was already practiced without official recommendations and underscores notable cross-cultural variation in the extent to which social interaction occurred. Results suggest that policy intervention should emphasize certain behavioral changes based on pre-existing context-specific patterns of interaction and interpersonal spacing rather than a one-size-fits-all approach. This research note is a descriptive first step that allows unique insight into social spacing and contact prior to the spread of SARS-CoV-2. It provides a baseline typology and a reference for future work on the cross-cultural implications of COVID-19 for pre-pandemic socio-cultural practice and vice versa.

Interpersonal spacing and social contact are core features of social interaction. They are also pathogen vectors, providing a bridge and an opportunity for transmission. As a result, public health interventions (e.g., physical distancing, limits on social contact) that limit the spread of SARS-CoV-2 also impact long-standing socio-cultural practice. We employ pre-pandemic cross-cultural data to assess how more than 20 countries navigated social interaction before the current pandemic, focusing on two dimensions: *interpersonal spacing*, which is measured using a unique data set of preferred distance from others (strangers, acquaintances and close contacts), and *social contact*, which is measured using survey data on frequency of religious service attendance and social gatherings (friends, relatives and colleagues).

*Interpersonal spacing* captures preferences in terms of physical distance when interacting with others. Variation has been observed between countries (Remland et al., 1995; Sorokowska et al., 2017), by gender (Ozdemir, 2008; Smith, 1981) and by age (Ozdemir, 2008; Webb & Weber, 2003). The circumstances of the interaction is key and notable variation is observed within a given context depending on the type of interaction, distinguishing those with close ties from strangers and acquaintances (Sorokowska et al., 2017). The metric used captures average preferences by country, measured in centimeters, in terms of spatial distance from three types of others: *stranger*, *acquaintance* and a person considered *close*.

*Social contact* refers to the frequency and nature of social interactions. As with interpersonal spacing, notable variation in the timing and extent of social contacts has been observed (Mossong et al., 2008). Variation by gender and socioeconomic characteristics are notable (Sayer, 2005) with implications of disease transmission (Kwok et al., 2014; Strömgren et al., 2017). Large gatherings, particularly religious services, are important forms of social interaction and have secondary implications for health and wellbeing (Nicholson, 2009). The metric used is derived from survey data that measures the frequency of *religious service attendance* and *social gatherings* with others defined as *friends*, *relatives* and *colleagues*.

## Data

Information on religious service attendance and frequency of social contact is recorded in the European Social Survey (ESS). The 9th and most recent wave (ESS9), collected in 2018, provides information for most countries (European Social Survey Round 9 Data 2018). For countries that did not participate in ESS9, the most recent available wave was used: Spain (ESS8), Greece (ESS5), Croatia (ESS5), Portugal (ESS8), Russia (ESS8), Slovakia (ESS6), Turkey (ESS4), Ukraine (ESS6) (European Social Survey Cumulative File 2018). For religious service attendance, respondents can reply on a 7-point ordinal scale ranging from [1] “never” to [7] “once a week”. The frequency of meetings with friends, relatives and colleagues is recorded on a 7-point scale ranging from [1] “never” to [7] “every day”. Responses of [1] “never” for religious services and [3] “once a month” for frequency of meetings with friends, relatives and colleagues are considered analogous to limited social interaction and avoidance of large gatherings.

Interpersonal spacing for three types of social engagement–strangers, acquaintances, and people with whom one is close–is measured in centimeters and was collected in 2013 (for complete details of the sample and method see Sorokowska et al., 2017). Distances of 100 cm are considered compliant with WHO’s suggested minimal separation of 1 m (World Health Organization, 2020) for effective social distancing. All participants were personally recruited through advertisements, personal contacts, in shopping malls to ensure the highest achievable proportion of community members in the final samples. The data collection was conducted during the same time across all locations and all participants were ensured anonymity. The original data includes 8943 participants from 53 study sites across 42 countries. Only European country contexts that offered equivalent samples from the ESS could be included in the analysis, resulting in a final sample of 22 countries. Appendix Table 1 presents basic demographic characteristics of the samples for countries included in the analysis. The complete replication package is available for download https://github.com/mathewcreighton/social_spacing/blob/0dd63e9e6dd671f56f964289dec4749b6a8590d7/Spatial_Demography_Research_Note_Replication_Packagev1.zip.

### Patterns of Pre-Pandemic Interpersonal Spacing, Religious-Service Attendance and Frequency of Social Contact Across Relationship Categories

**Fig. 1:**
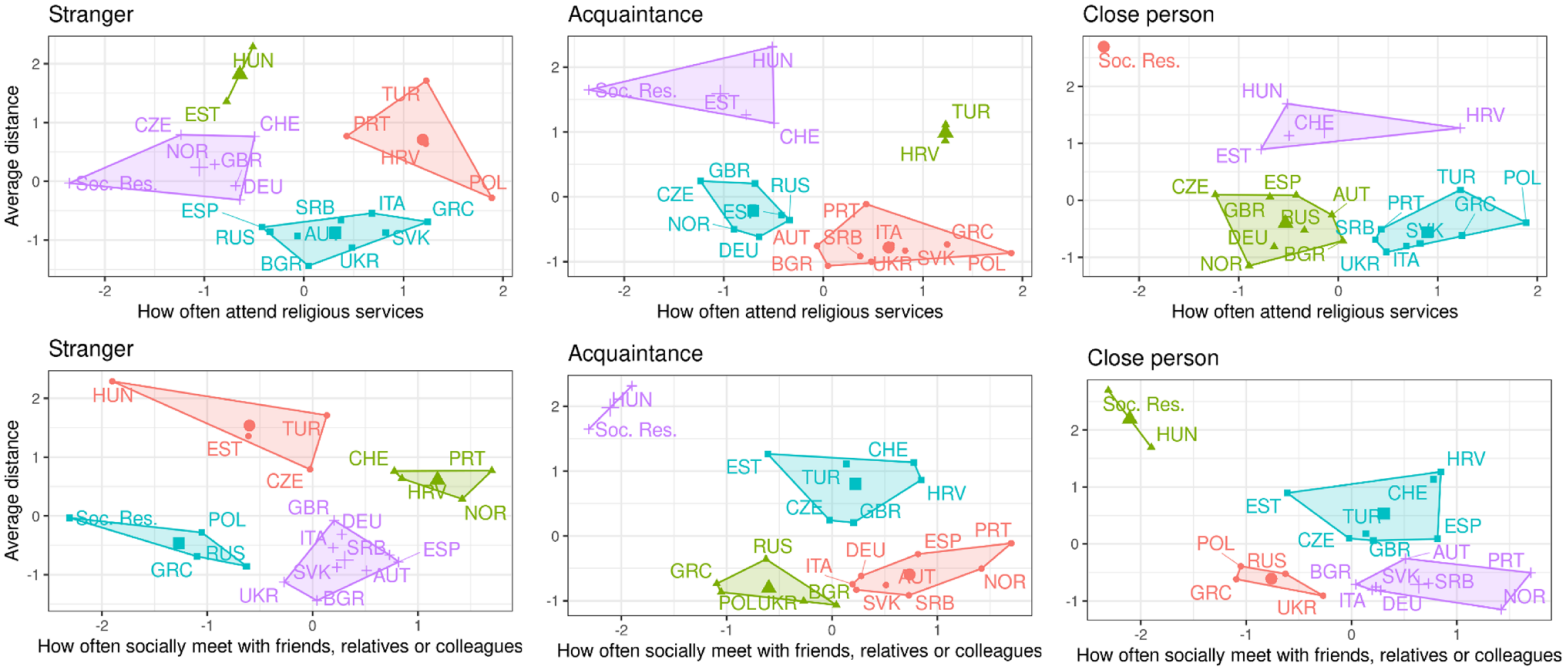
Relationships between social contact, religious service attendance and interpersonal spacing. (*The clustered scatter plots show pre-pandemic comparisons of social contact, religious service attendance and interpersonal spacing (preferred distance from strangers, acquaintances and close friends). The scale is standardized and reports z-scores for the measures on the y-axis and x-axis. On both axes, higher values indicate greater interpersonal spacing and more frequent social interaction. A hypothetical context, labelled “Soc. Res.”, indicates a socially restricted context with average interpersonal spacing maintained in accordance with WHO recommendations (100 cm) and limited social contact (meeting friends once a month and no religious service attendance). Similar groupings of countries are encircled and highlighted in the same color. The complete replication package is available for download* https://github.com/mathewcreighton/social_spacing/blob/0dd63e9e6dd671f56f964289dec4749b6a8590d7/Spatial_Demography_Research_Note_Replication_Packagev1.zip)

Figure 1 shows pre-pandemic estimates of social contact, religious service attendance and average preferred interpersonal spacing by country. K-means clustering is used to define similar groupings by placing n observations into k clusters. The number of clusters was determined through the "elbow method" in which the optimal k was selected when an additional number of clusters would not improve the partition of the total within-cluster sum of squares (WSS). The algorithm used to assign the observations into clusters (Hartigan & Wong, 1979) includes three steps. The algorithm begins by selecting randomly k observations as the preliminary centroid of the cluster. After that, the remaining observations are assigned to their closest centroid. Then a new centroid is generated based on the mean value of each cluster. The second and third steps are repeated until it reaches a minimum within-cluster sum of squares. Figure 1 then shows country clusters where the centroid is the mean of the z-scores for physical distance (Y) and frequency of contact (X).

Consistent with other work (Sorokowska et al., 2017), interpersonal spacing declines notably when the interaction includes people with stronger social ties, therefore the analyses were performed separately for strangers, acquaintances and friends. For comparative purposes, we also included the hypothetical context labelled “Soc. Res.”, which is defined as the absence of religious gatherings, infrequent social contact (i.e., once a month or less) and 100 cm social spacing, which conforms with the World Health Organization’s (WHO) suggested minimal guidelines (World Health Organization, 2020). Although we consider the WHO’s 100 cm suggested minimal distance to be a reasonable reference for international comparative work, 6ft or 200 cm is often used by national and international bodies as the preferred distance to minimize the risk of transmission.

As illustrated in Fig. 1, Hungary, Turkey, Estonia, Czech Republic, Portugal, Norway, Croatia, Great Britain, Germany and Switzerland report a preferred distance near or in excess of WHO guidelines for interactions with strangers. For acquaintances, only Hungary and Estonia prefer 100 cm + on average. Most counties prefer distances below 100 cm on average in all situations.

Patterns of religious service attendance reveal the multidimensionality of behavior. Some countries that report, on average, greater interpersonal spacing as preferable (e.g., Turkey, Portugal, Croatia) are also among the most frequent attenders of religious services. Only Hungary situates itself nearer to a socially restrictive context in terms of both interpersonal spacing and attendance of religious services. Of note, countries that prefer closer interpersonal spacing, regardless of category (e.g., Greece, Russia and Poland) are notably infrequent in terms of social contact.

### Clusters of Pre-Pandemic Interpersonal Spacing, Religious-Service Attendance and Frequency of Social Contact

**Fig. 2:**
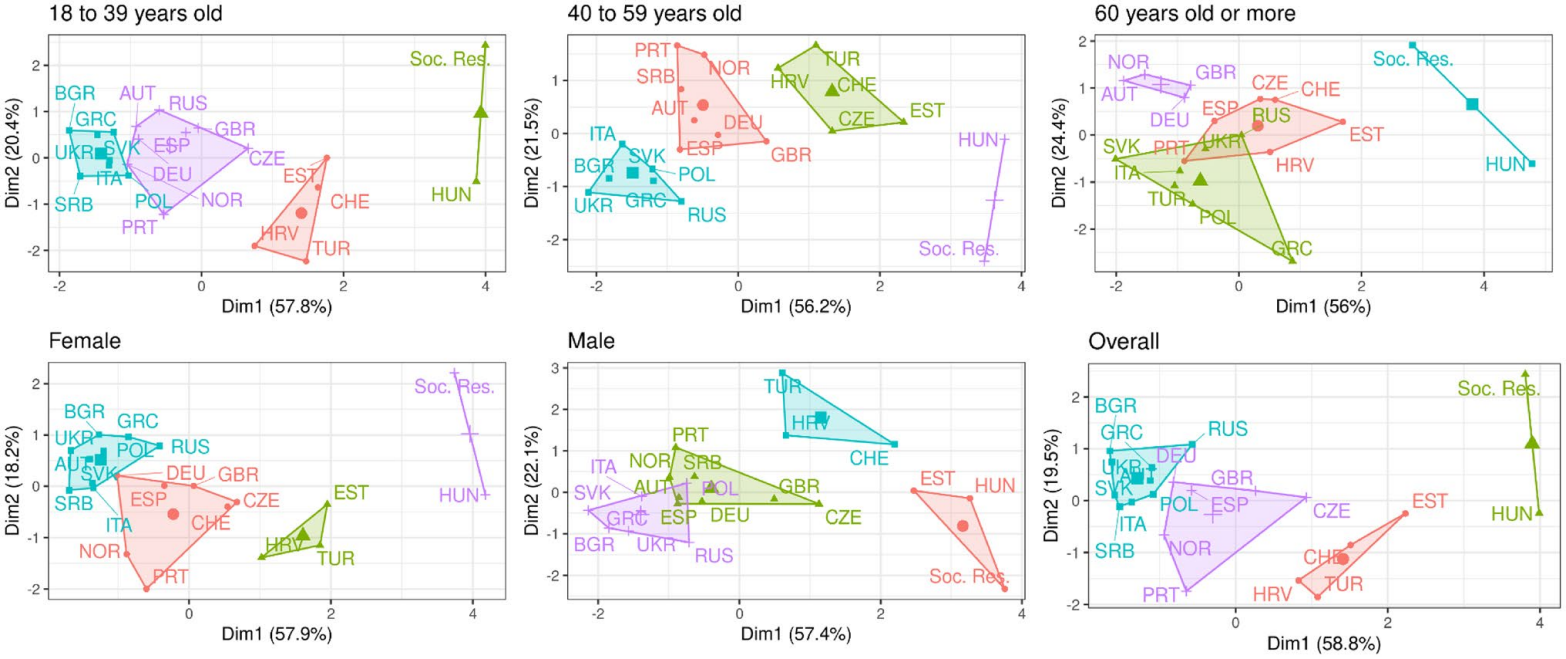
Cluster analysis of social contact, religious service attendance and interpersonal spacing by age, sex and overall (*The cluster analysis shows pre-pandemic measures of social contact, religious service attendance and interpersonal spacing (preferred distance from strangers, acquaintances and close friends) by age, sex and overall. To present the analysis, standardized cluster plots are used the report two scaled dimensions of the cluster analysis. The x-axis and y-axis indicate relative position on scales derived from first and second order dimensions in terms of the extent to which within-group variation is explained. A hypothetical context, labelled “Soc. Res.”, indicates a socially restricted context with average interpersonal spacing maintained in accordance with WHO recommendations (100 cm) and limited social contact (meeting friends once a month and no religious service attendance), which was included in the estimation of the clusters. Similar groupings of countries are encircled and highlighted in the same color. The complete replication package is available for download* https://github.com/mathewcreighton/social_spacing/blob/0dd63e9e6dd671f56f964289dec4749b6a8590d7/Spatial_Demography_Research_Note_Replication_Packagev1.zip)

Figure 2 reports k-mean cluster analysis that includes social interaction (religious service attendance and frequency of social contact) and all dimensions of preferred interpersonal spacing–stranger acquaintance, close tie). The approach used to generate Fig. 2 is similar to that used in Fig. 1’s bivariate plots, but instead of only two variables, all measures of social contact, religious service attendance and interpersonal spacing are used. The axes represent the two first dimensions obtained through principal component analysis and, in parenthesis, the percentage of variance explained by the referred dimension. The analysis can be reproduced through the publicly available replication package which contains the working datasets, the R Code, the "elbow method" figures and cluster figures. From this descriptive analysis,^1^ three broad categories emerge that reflect a pre-pandemic patterns to social spacing and interaction.

First, we find a group of countries reflect *limited spacing* in that, without clear advice on interpersonal spacing, often termed social distancing although physical distancing is more accurate (Das Gupta and Wong, 2020; Gale, 2020; Sørensen et al., 2021), and limits to social contact, there is a natural tendency toward close and frequent contact. Italy, Bulgaria, Serbia, Ukraine, Slovakia and Greece are exemplary. In these cases, social distancing and limitations on social contact would entail a relatively more significant socio-behavioral shift.

Second, we have countries that cluster somewhat closer to a recommended, socially restrictive context, termed “Soc. Res.”. These contexts offer *mixed spacing*. This cluster includes countries like Spain, Great Britain and Germany and contrasting patterns in terms of social interaction and interpersonal spacing. For example, religious service attendance is infrequent (e.g., Spain, Great Britain), but preferred physical distance is relatively close. These contexts would be best served by targeted policy interventions that emphasize country-specific areas of concern, which could focus on physical distance or social contact/gatherings or a more nuanced variation.

Third, we have countries offer *natural spacing* in that they that cluster close to (and occasionally within) a “safe” hypothetical context practicing 1-m social distancing, prohibiting religious gatherings and experiencing infrequent social contact. Turkey, Switzerland, Croatia and Estonia fall into this category. A fourth cluster, consisting only of Hungary, is closest to WHO guidelines on social distancing and limits on social contact/gathering without intervention. This is not to say that transmission of SARS-CoV-2 is unlikely or even less likely to occur in these contexts. Instead, the pattern suggests that conforming to socially restrictive guidelines would not imply as large a socio-behavioral shift.

### Patterns by Age and Sex

The transmission of SARS-CoV-2 and the health consequences of COVID-19 vary by sex and age with men and older people disproportionately and negatively affected (Dowd et al., 2020). Figure 2 underlines two patterns. First, there is some natural movement toward more social restriction as age increases. The overall pattern remains largely true for the older ages as well with some exceptions. For example, Turkey shifts further from social restriction as age increases, suggesting that those aged 60 + are relatively more socially active. Second, there is a clear pattern by sex with males gravitating toward a more socially restricted position. Estonia and Hungary, men share more with a context experiencing near-total social restriction than anywhere else.

### Implications For Policy

Adapting to a post-pandemic “new normal” often requires deviating from life-long behavioral practices that govern how we socially interact. Policy interventions that ask for social distancing, prohibit religious gatherings and dictate infrequent social contact impact everyone, but not equally. This research note provides unique insight into the social context preceding the outbreak of COVID-19 in 20 countries. As work on Ebola underscored, socio-cultural behavior is an important factor to understand when modelling transmission patterns (Chowell & Nishiura, 2014). Some countries naturally engage in greater protective behavior and would require relatively limited shifts in social behavior due to SARS-CoV-2. The most obvious example is Hungary, where interpersonal spacing, social contact and religious gatherings are notably compatible with post-pandemic social restrictions. In other contexts, the opposite is true and limits on physical distance and contact would entail a relatively large deviation from ongoing social practice. These less socially restrictive contexts would plausibly benefit from greater supports that underscore how and when one should deviate from long-standing forms of socializing. Other contexts are mixed and one type of intervention (e.g., limits on social contacts) would require less adaptation relative to others (e.g., close interpersonal spacing with strangers).

This research note is a descriptive, but crucial first step toward understanding social contact, interaction and interpersonal spacing prior to the spread of SARS-CoV-2. Next steps would be to consider the link between pre-pandemic physical distance/contact for other demographic patterns such as re-infection rates, mortality, and long-term compliance with socially restrictive public health measures. In addition, links between natural patterns of interpersonal spacing and contact and transmission rates during annual rituals (e.g., religious holidays, summer vacations, etc..) deserve greater scrutiny. What is clearly highlighted in this work is that long-standing socio-cultural practice varies significantly between country contexts. Moreover, this variation is directly linked to public health mitigation strategies put in place to mitigate the transmission of SARS-CoV-2 (e.g., guidance on physical distancing, mandated restriction on public gatherings like religious services). As a result, any future analysis of post-pandemic shifts in these same practices, including that which explores how public health measures shape short- and long-term social relationships, requires pre-pandemic baseline levels of social contact, interaction and interpersonal spacing to be taken into account.

## Appendix

See Table 1.

**Table 1:** Demographic characteristics for each sample of interpersonal spacing

Country	Sample size			Age	
	Total	Men	Women	*M* (*SD*)	Range
AUT	200	115	85	26.59 (9.73)	17–65
BGR	102	63	39	38.35 (8.95)	21–59
HRV	614	301	313	44.75 (11.65)	19–83
CZE	167	80	87	36.48 (15.93)	18–79
EST	149	50	96	42.93 (12.30)	20–74
DEU	154	62	92	31.59 (13.39)	18–74
GRC	94	42	49	38.77 (9.07)	20–71
HUN	237	76	161	37.80 (9.56)	19–62
ITA	322	127	195	48.39 (11.06)	20–86
NOR	100	72	28	41.29 (13.51)	22–77
POL	428	161	254	40.07 (11.66)	20–87
PRT	293	99	181	46.04 (11.17)	18–81
RUS	224	120	104	38.61 (13.86)	19–87
SRB	105	19	86	24.96 (7.01)	20–56
SVK	233	76	157	42.76 (11.74)	22–72
ESP	199	93	106	47.10 (9.36)	24–67
CHE	179	110	69	48.77 (12.87)	21–75
TUR	391	238	153	42.70 (13.59)	20–83
GBR	100	42	58	45.04 (11.57)	20–78
UKR	311	66	245	29.20 (8.73)	18–61

*This table is adapted from *Sorokowska et al., 2017* and shows the core demographic characteristics of the sample used to estimate country-level interpersonal spacing preferences in centimetres. Age ranges differences reflect the feasibility of the data collection in each country context. Complete details about the data collection and initial measurement can be found in *Sorokowska et al., 2017*. The complete replication package is available for download* https://github.com/mathewcreighton/social_spacing/blob/0dd63e9e6dd671f56f964289dec4749b6a8590d7/Spatial_Demography_Research_Note_Replication_Packagev1.zip)

## Footnotes

1 K-means clustering does not take into account the error in the mean for a given dimension. Although informative, the clusters that emerge are best interpreted as descriptive and this research note offers a pathway toward further assessment of pre-pandemic socio-behavioral context with more robust measures of social contact and interpersonal spacing.
